# The value of left atrial longitudinal strain in evaluating left atrial appendage spontaneous echo contrast in non-valvular atrial fibrillation

**DOI:** 10.3389/fcvm.2023.1090139

**Published:** 2023-07-06

**Authors:** Chang sheng Ma, Shi kun Sun, Li Wang, Bing yuan Zhou, Feng lin Dong

**Affiliations:** Department of Cardiology, The First Affiliated Hospital of Soochow University, Suzhou, China

**Keywords:** spontaneous echo contrast (SEC), non-valvular atrial fibrillation, left atrial reserve function, strain, correlations, left atrial appendage

## Abstract

**Background:**

Spontaneous echo contrast (SEC) observed in transesophageal echocardiography (TEE) is a reliable predictor of the risk of future ischemic stroke in patients with non-valvular atrial fibrillation (NVAF). Left atrial strain globally reflects atrial function, remodeling and distensibility. The left atrial appendage (LAA) is a myogenic remnant of the left atrium, which can actively relax and contract. The left atrial appendage (LAA) is an important part of releasing the pressure of the left atrium. The key role of the left atrium is to regulate the left ventricular filling pressure, act as a reservoir for pulmonary venous return during ventricular contraction, and act as a conduit, transferring blood to the Left ventricle during early ventricular diastole. The purpose of this study was to explore the relationship between left atrial function and left atrial appendage spontaneous echo contrast (LAASEC).

**Methods:**

A retrospective study of 338 patients with non-valvular AF was conducted. Two-dimensional speckle-tracking echocardiography provided the following metrics of LA strain: LA strain during the reservoir phase (LASr), LA strain during the conduit phase (LAScd). LA or LAA has the dense SEC of more than grade 3, which is defined as mud like change or pre thrombosis.

**Results:**

Patients with level 3 SEC (*n* = 81) has lower LASr than those with lower grades of SEC (*n* = 257) (7.20 ± 3.70 vs. 17.48 ± 8.67, *P* < 0.001). Multivariate logistic regression model showed that the type of atrial fibrillation (persistent∼), increased heart rate, decreased LASr were independently associated with the dense LAASEC (OR (CI 95%), 5.558 (1.618–19.09), 1.016 (1.006–1.026) 0.002, 1.224 (1.085–1.381), both *P* < 0.01). Venn Diagram showed that lower CHADVASC2 score groups had dense SEC cases. Receiver operating characteristic (ROC) curve was used for analyzing results and selecting cut off values. The cut off point for LASr < 8.85% and CHADVASC2 score was >2 scores with sensitivity and specificity were 79% and 85%.

**Conclusion:**

Lower LASr is independently associated with the dense LAASEC in NVAF and has incremental values superior to clinical scores. The decrease of LASr may be a potential non-invasive parameter for evaluating the higher risk of LAA thrombosis.

## 1. Introduction

Atrial fibrillation (AF) is the most common arrhythmia effecting up to 2% of the population (1). Previous studies have shown that these patients were characterized by higher stroke rate, higher thromboembolic events, left ventricular dysfunction and heart failure. At present, the prevalence rate of atrial fibrillation is as high as 1.1% in China, showing a significant growth trend (1, 2). Left atrial appendage (LAA)/left atrial (LA) thrombosis is considered the main source of systemic thrombosis in patients with AF (3). More than 75% of ischemic stroke in patients with AF was due to thromboembolism located in the LA or LAA (3). Some studies have reported the potential relationship between LA structure and the risk of ischemic stroke in patients with AF (4–6). Clinical scores are commonly used to predict the risk of stroke and systemic embolism in patients with AF at present. This CHADVASC2 and CHADS2 are anticoagulant scoring system in clinical practice. Although subsequent studies have also successively proposed other clinical scores, such as The Four Thromboembolism Risk Factors and The Framingham Heart Study scoring system, the common shortcomings of these scores are only applicable to the general risk factors of stroke assessment. However, stroke risks in patients with NVAF is usually related to thromboembolism caused by cardiogenic thrombosis, and these scores are not included in the structural/functional information of LA/LAA, that is, the hypothetical description of cardiogenic embolism risk is not enabled. Spontaneous echocardiographic contrast (SEC) and thrombus observed in transesophageal echocardiography (TEE) are considered as powerful surrogate markers for the risk of future ischemic stroke in patients with atrial fibrillation or atrial flutter (7). The correlation between the transesophageal echocardiography (TEE) and CHADS2 or CHADVASC2 scores in patients with NVAF showed that the higher CHADS2 and CHADVASC2 scores, the greater the possibility of observing left atrium/left atrial appendage spontaneous echo contrast (LASEC/LAASEC) (8); and that, despite it became lower, a certain number of patients were found as usual to have LASEC/LAASEC (9) The CHADVASC2 [congestive heart failure, hypertension, age ≥75 years, diabetes mellitus, stroke or transient ischemic attack (TIA), vascular disease, age 65–74 years, sex category] score is the most common method for stratifying thromboembolism risk in patients with AF, and it is also used to inform decisions on anticoagulation therapy. At present, there are few studies on the clinical relationship between LA and LAA. This study mainly explores the correlation between LA function and LAA blood flow status.

## 2. Method

A total of 338 patients with non-valvular atrial fibrillation who were admitted to the Department of Cardiology of the First Affiliated Hospital of Suzhou University from December 1, 2018 to June 10, 2021 were included in the retrospective study. 338 patients with AF included in the study were prepared to undergo radiofrequency catheter ablation. All patients were examined by 12-lead electrocardiogram or 24-hour dynamic electrocardiogram, and the onset of AF was clearly recorded. All of them received Transthoracic echocardiography (TTE) and Transesophageal echocardiography (TEE). Paroxysmal atrial fibrillation was defined as AF can recover spontaneously within 7 days or under intervention, and recur at different frequencies; Persistent atrial fibrillation refers to the onset time of continuous AF >7 days. The inclusion criteria were as follows: patients with non-valvular atrial fibrillation (NVAF) with adequate image quality and complete clinical data. Informed consent was obtained from each patient. Basic information collected about the patients included age, height, weight, and the type of AF (paroxysmal or persistent). Previous history of hypertension, diabetes, coronary heart disease, prior stroke, or congenital heart disease was recorded. The exclusion criteria were as follows: patients with congenital heart disease, valvular heart disease, thrombocytopenic purpura, severe mitral regurgitation, or tumor, and patients whose data included low-quality images. All patients must undergo TEE and TTE before treatment, each participant signed a written informed consent form. This study was conducted in accordance with the Declaration of Helsinki (as revised in 2013) and approved by the Ethics Committee of The Affiliated Hospital of Soochow University (No. 225/2022).

### 2.1. TTE

Vivid E95 ultrasonic system (GE E95, Norway) equipped with M5S probe was used for imaging. This study was performed in the left recumbent position, connecting with ECG. We selected cardiac cycles with minimal differences and took the average heart rate when obtained images, especially for per-AF. Standard two-dimensional echocardiographic views were obtained, including parasternal long axis and apical four chamber views and apical two chamber views. The Biplane Simpson' method was used to obtain the maximum and minimum left atrial (LA) volumes of the apical four chamber view and the apical two chamber view, and the exponential standardization was performed according to the Body Mass Index (BMI) to calculate the total left atrial emptying fraction (LAEF). The early diastolic flow-velocity peak of the mitral valve (peak E) was measured by pulse Doppler in the apical four-chamber view. Doppler Tissue Imaging (DTI) was used to measure the early diastolic tissue Doppler velocity of the mitral annulus from septal and lateral (E'sep and E'la). The ratio of E to E' (E/e') was then calculated. Simpson's rule was used to measure the left ventricular ejection fraction (LVEF) in the apical four-chamber view and the apical two-chamber view. The method of evaluating cardiac function according to the current guidelines of the American Society of Echocardiography (10).

### 2.2. TEE

**Figure 1:**
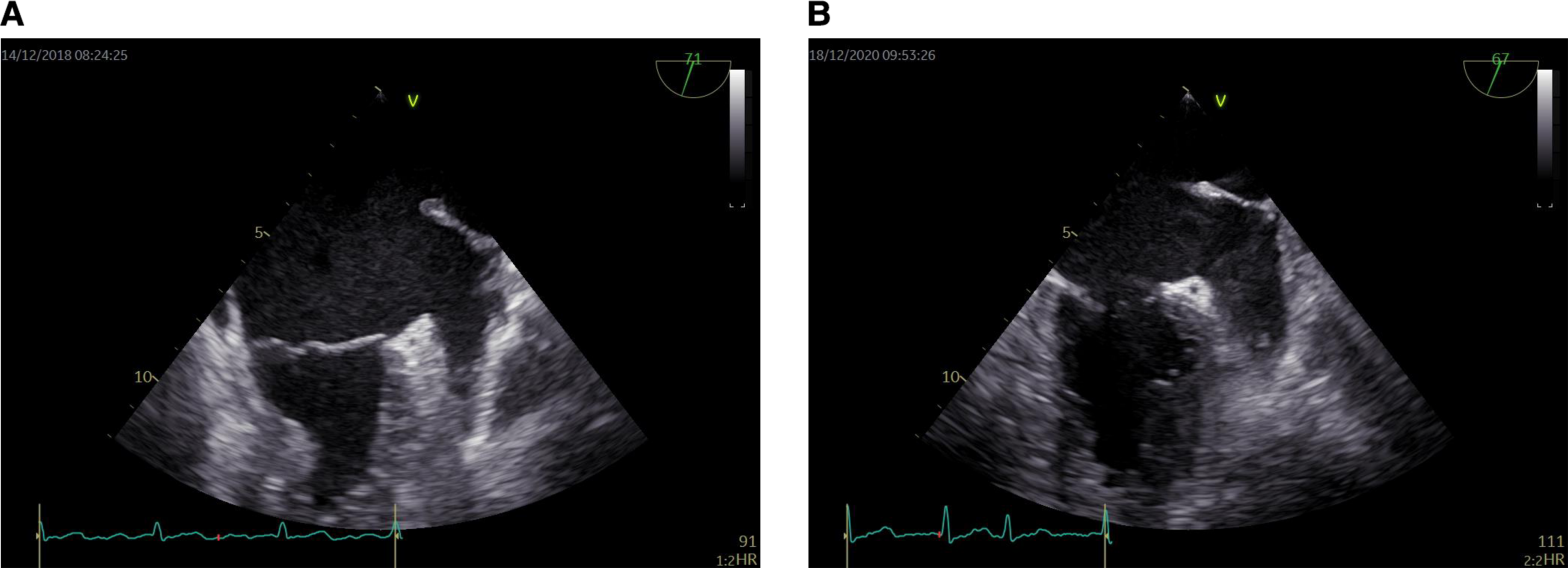
Dense LAASEC. (**A**) Level 3 LAASEC. (**B**) Level 4 LAASEC. LAASEC, left atrial appendage spontaneous echo contrast.

All patients routinely used Vivid E95 echocardiography for TEE, equipped with multiplane 6VT (3.0–8.0 MHz) transducers. All TEE assessments were performed by professional physicians at least different views (45°, 60°, 90°, 135° views of the middle esophagus) should be used to evaluate the left atrial appendage (LAA). The LAA were evaluated comprehensively to find any evidence of SEC or thrombus. During left atrial appendage imaging, pulse Doppler velocity of left atrial appendage forward (emptying) and backward (filling) blood flow was also recorded. Before TEE, lidocaine hydrochloride spray was used for local anesthesia of throat. The left atrial appendage was evaluated from 0° to 180° in the middle of the esophagus. Left atrial appendage ejection fraction (LAAEF) was measured and calculated by 2D Simpson' s method. (LAAEF) = [LAA Vmax—LAA Vmin]/LAAVmax × 100%. When the left atrium or left atrial appendage has the dense SEC of more than grade 3, it will show a jelly like non solid shape that lasts throughout the cardiac cycle and has a tendency to deposit, which is defined as mud like change or pre thrombosis (11, 12). SEC of LAA (Figure 1).

### 2.3. LA 2D-STE analysis

**Figure 2:**
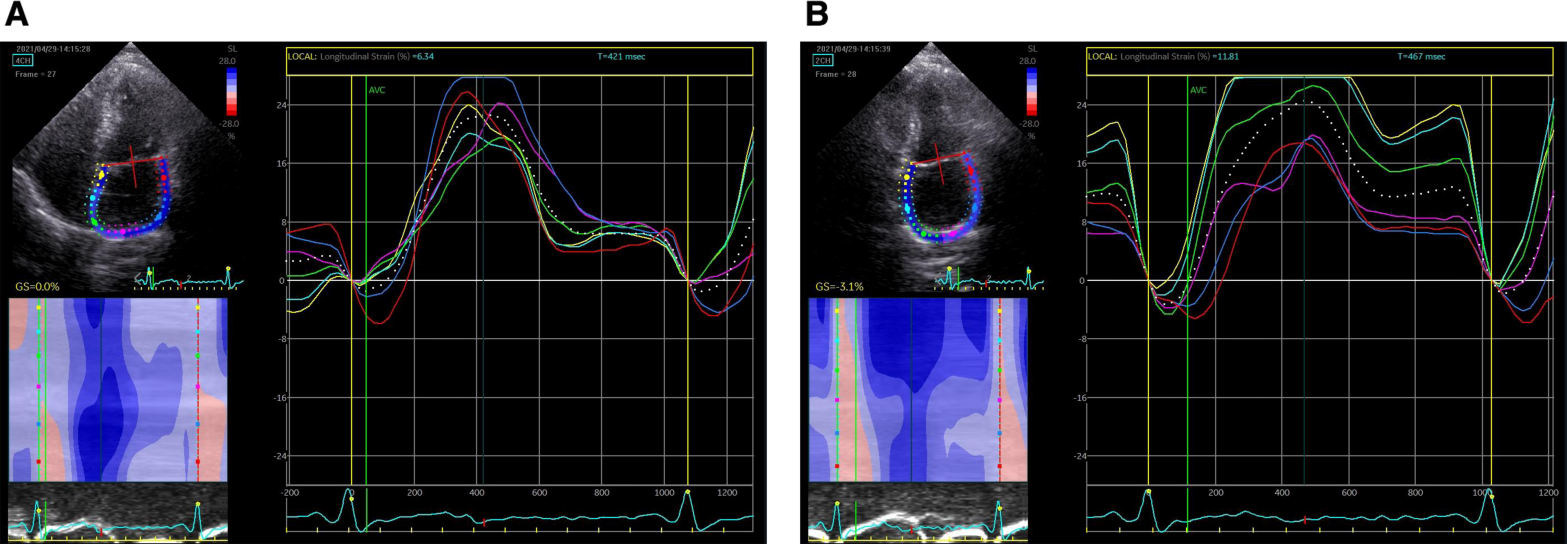
Left atrial strain curves. (**A**) Left atrial strain curves in the four chamber view; (**B**) left atrial strain curves in the two chamber view.

EchoPAC (GE Medical Systems) has been used in most studies on the deformation of left atrium (LA) previously, with high feasibility and good consistency. Speckle tracking echocardiography was performed using offline analysis (201 version). The starting point of the QRS wave of in the (electrocardiogram)ECG was used as the zero baseline. 2D-STE is a speckle tracking analysis technique using standard B-mode images. During brief breath holding and stable ECG recording, conventional 2D gray scale echocardiography was used to obtain apical four chamber and two chamber views. Patients with atrial fibrillation measure almost five consecutive cardiac cycles on average, find out and record three average consecutive cardiac cycles. The recommended frame rate setting is 60–80 frames per second. During processing, the left atrial endocardial surface was manually tracked by tracing in two chamber and four chamber views. In order to obtain the region of interest (ROI), the system automatically generates atrial endocardial surface tracking. You can manually adjust the width and shape of the ROI, and then the software divides it into six parts, and the tracking quality of each part is automatically adjusted. For each segment, the software gives the longitudinal strain curve and the average curve of all segments reflecting the pathophysiology of left atrial function. If more than three segments were excluded, subjects were removed from the study. The left atrial strain curves (Figure 2). The left atrial peak longitudinal strain (LASr) during ventricular contraction and the left atrial longitudinal strain (LAScd) during early diastole were measured. These values were taken as the average of the four chamber view and the two chamber view.

Repeatability evaluation of measurement parameters.

Two imaging cardiologist analyzed the image in the EchoPAC softwear after obtained. Each researcher analyzed the image twice to avoid the intra-group differences. And take the average value of the data collected by two researchers as the final data to avoid inter-group differences.

## 3. Statistics

Continuous variables of normal distribution were expressed as mean ± standard deviation. The variable of unnormal distribution was expressed as the median (quartile range), and the categorical variable was expressed as the frequency (percentage). The *t*-test or Mann Whitney *U*-test was used to compare the continuous variables between groups. The *χ*^2^-test or Fisher exact test were used to compare the categorical variables between groups. Single factor and multiple factors logistic regression analysis were conducted to determine the independent risk factors of LAASEC. In univariate analysis, variables with *P* < 0.05 were included in multivariate analysis. Subsequently, variables with *P* < 0.05 in multivariate analysis were included in the final multiple logistic regression model. Finally, Receiver operating characteristic (ROC) curve analysis was conducted to evaluate the predictive ability of left atrial function indicators and CHADVASC2 scores. Statistical significance was defined as *P* value <0.05. SPSS software (version 27.0) was used for statistical analysis.

## 4. Result

(1) A total of 338 patients with atrial fibrillation were included, 40.2% of whom were female, with an average age of 63.20 ± 10.36. 81 patients (23.96%) with LAASEC above grade 3, all patients' characteristics were recorded in Table 1. Among them, 57.7% were patients with persistent atrial fibrillation, 59.8% had hypertension, 9.8% had heart failure, and 14.2% had diabetes. 9.8% of the patients had experience of stroke before going to hospital.
(2) According to the level of LAASEC, they were divided into two groups (Table 1): those above level 3 (including level 3) were classified into one group, and those below level 2 (including level 2) were classified into the other group. There was no significant difference in hypertension history, Previous-Stroke, and systolic blood pressure between the two groups. The age of the group LAASEC grade 3 and above vs. the group LAASEC grade 2 and below was higher (66.17 ± 8.12 vs. 62.28 ± 10.82, *P* = 0.003), the proportion of women was higher (53.1% vs. 36.2%, *P* = 0.009), and the proportion of persistent atrial fibrillation was higher (91.4% vs. 47.1%, *P* < 0.001), Higher incidence of heart failure (26.3% vs. 4.7% *P* < 0.001), higher diastolic pressure (84.40 ± 11.91 vs. 81.39 ± 11.10, *P* = 0.038), higher heart rate (116.89 ± 39.13 vs. 97.08 ± 30.36, *P* < 0.001), higher CHADVASC2 score (2.68 ± 1.45 vs. 1.90 ± 1.44, *P* < 0.001), larger left atrial size (48.48 ± 4.68 vs. 43.54 ± 5.89, *P* < 0.001), diastolic left atrial volume index and systolic left atrial volume index were larger (64.23 ± 15.52 vs. 46.12 ± 16.05, 55.08 ± 15.35 vs. 31.08 ± 16.35, *P* < 0.001), Left ventricular ejection fraction and left atrial emptying fraction decreased (52.71 ± 11.84 vs. 60.94 ± 6.84, 8.92 ± 5.2 vs. 14.92 ± 6.98, *P* < 0.001), LASr and LAScd were lower (7.20 ± 3.70 vs. 17.48 ± 8.67, 6.92 ± 3.03 vs. 12.06 ± 5.54 *P* < 0.001), The LAA ostia and LAA depth were larger (20.62 ± 4.77 vs. 18.71 ± 3.58, 30.69 ± 6.77 vs. 27.05 ± 6.24, *P* < 0.001), LAAEV and LAAFV were lower (28.75 ± 10.25 vs. 48.94 ± 23.27, 32.36 ± 16.47 vs. 49.35 ± 19.70, *P* < 0.001), and LAAEF was decreased (32.52 ± 14.12 vs. 67.62 ± 25.29, *P* < 0.001).
(3) Factors associated with LAASEC

Univariate logistic regression identified a variety of clinical TTE, TEE parameters as significant contributors to LAASEC (Table 2): Age, Women, Persistent Atrial Fibrillation, Heart Failure, Diabetes, Higher heart rate, Higher CHADVASC2 scores, Increased left atrium and left atrial volume, Higher E/e'. On multi-variate analysis independent risk factors were persistent atrial fibrillation, higher heart rate, decreased LASr, Higher E/e' reminded to LAASEC [AF, (per-), 1.618–19.09, *P* = 0.006; decreased LASr 1.085–1.381, *P* = 0.001, higher heart rate, 1.006–1.026, *P* = 0.002].

**Figure 3:**
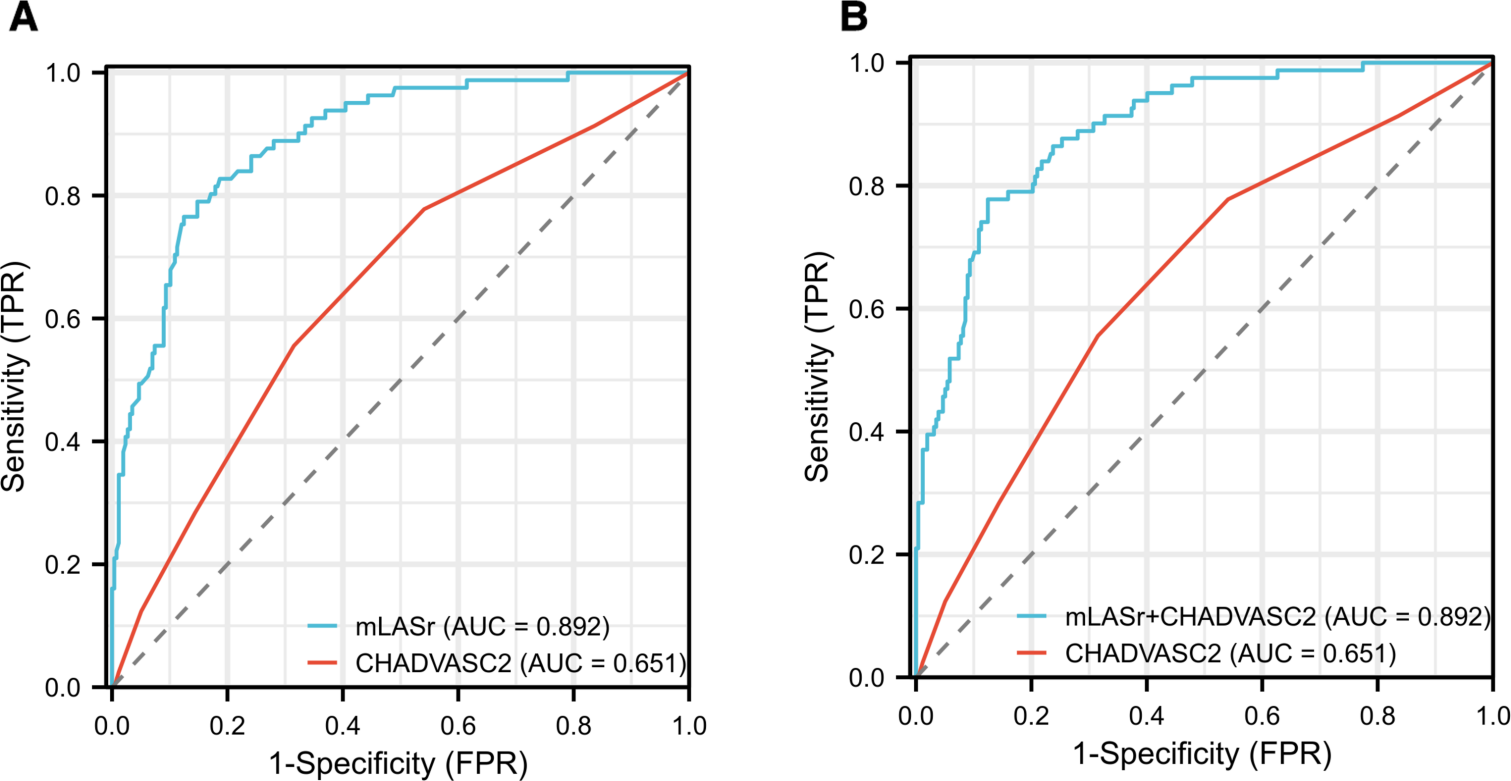
Results of receiver-operating characteristic curve analysis for identifying dense LAASEC. (**A**) The initial diagnosis model based on CHADVASC2 was improved by the mLASr. (**B**) The initial diagnosis model based on CHADVASC2 was improved by the mLASr + CHADVASC2. AUC, area under the curve; LAA, left atrial appendage; mLASr, left atrial peak longitudinal strain.

(4) The ROC curve analysis results showed (Figure 3):

**Table 1:** Baseline demographics of the population.

	Total (*n* = 338)	Lower SEC (*n* = 257)	Higher SEC (*n* = 81)	*P*
Age, years, Y	63.20 ± 10.36	62.28 ± 10.82	66.17 ± 8.12	0.003
Sex, woman, %	40.2	36.2	53.1	0.009
AF, (per-)	57.7	47.1	91.4	<0.001
Hypertension, *n*, %	59.8	58.4	64.2	0.366
Diabetes, *n*, %	14.2	11.3	23.5	0.010
Previous-stroke, *n*, %	9.8	9.7	9.9	1.000
Heart failure, *n*, %	9.8	4.7	26.3	<0.001
Systolic pressure, mmHg	128.11 ± 15.99	128.46 ± 15.57	127.00 ± 17.34	0.474
Diastolic pressure, mmHg	82.11 ± 11.35	81.39 ± 11.1	84.40 ± 11.91	0.038
heart rate, bpm	101.86 ± 33.71	97.08 ± 30.36	116.89 ± 39.13	<0.001
left atrium, mm	44.71 ± 6.0	43.54 ± 5.89	48.48 ± 4.68	<0.001
LVEF, %	58.99 ± 8.99	60.94 ± 6.84	52.71 ± 11.84	<0.001
LAVI (diastolic), ml/m^2^	50.40 ± 17.67	46.12 ± 16.05	64.23 ± 15.52	<0.001
LAVI (systole), ml/m^2^	36.75 ± 19.06	31.08 ± 16.35	55.08 ± 15.35	<0.001
LAEF, %	13.49 ± 7.07	14.92 ± 6.98	8.92 ± 5.20	<0.001
LARs, %	15.01 ± 8.93	17.48 ± 8.67	7.20 ± 3.70	<0.001
LASct, %	10.83 ± 5.51	12.06 ± 5.54	6.92 ± 3.03	<0.001
Cha2ds2vasc	2.10 ± 1.45	1.90 ± 1.44	2.68 ± 1.45	<0.001
E (cm/s)	85.37 ± 21.98	82.34 ± 22.13	95.11 ± 18.50	<0.001
EDT (s)	187.36 ± 53.05	195.01 ± 52.74	159.04 ± 44.16	<0.001
E‘ (sep) (cm/s)	7.55 ± 2.32	7.73 ± 2.37	6.97 ± 2.06	0.011
E’(la) (cm/s)	10.50 ± 3.30	10.63 ± 3.53	10.09 ± 2.43	0.216
E/e’	10.14 ± 4.01	9.64 ± 3.80	11.77 ± 4.30	<0.001
PASP (mmHg)	27.93 ± 6.55	26.43 ± 6.19	31.19 ± 9.46	<0.001
LAAEV, cm/s	43.32 ± 23.10	48.94 ± 23.27	25.78 ± 10.25	0.008
LAAFV, cm/s	45.23 ± 20.30	49.35 ± 19.70	32.36 ± 16.47	<0.001
LAA ostia, mm	19.17 ± 3.98	18.71 ± 3.58	20.62 ± 4.77	<0.001
LAA depth, mm	27.93 ± 6.55	27.05 ± 6.24	30.69 ± 6.77	<0.001
LAAEF, %	59.13 ± 27.55	67.62 ± 25.29	32.52 ± 14.12	<0.001

LVEF, Left ventricular ejection fraction; E, The early diastolic flow-velocity peak of mitral valve; (E'sep and E’la), early diastolic tissue Doppler velocity of mitral annulus from septal and lateral; E/e’, ratio of peak early mitral inflow velocity and peak early diastolic mitral annular velocity; PASP, pulmonary artery systolic pressure; LAAEV, the peak empty velocity of left atrial appendage; LAAFV, the peak filling velocity of left atrial appendage; LAA, left atrial appendage; LAAEF, Left atrial appendage ejection fraction; LARs, left atrial reservoir strain; LASct, left atrial strain conduit; LAVI, Left atrial volume index.

**Table 2:** Univariable and multivariable logistic regression analysis of between clinical and echocardiographic covariates with dense SEC.

	Univariable logistic regression		Multivariable logistic regression	
	OR (95% CI)	*P*	OR (95% CI)	*P*
Clinical parameters				
	1.043 (1.014–1.073)	0.004		
Sex, woman, %	1.995 (1.204–3.307)	0.007		
AF, (per-)	11.88 (5.271–26.785)	0.000	5.558 (1.618∼19.09)	0.006
Diabetes, *n*, %	2.409 (1.267–4.583)	0.007		
Heart failure, *n*, %	7.267 (3.385–15.602)	0.000		
heart rate, bpm	1.017 (1.009–1.024)	0.000	1.016 (1.006–1.026)	0.002
Cha2ds2vasc	1.416 (1.193–1.680)	0.000	1.271 (0.998–1.620)	0.052
LA parameters				
Left atrium, mm	1.195 (1.130–1.263)	0.000		
LVEF, %	0.905 (0.877–0.934)	0.000	1.051 (1.003–1.100)	0.038
LAVI (diastolic), ml/m^2^	1.063 (1.045–1.081)	0.000		
LAVI (systole), ml/m^2^	1.084 (1.063–1.105)	0.000	1.140 (1.060–1.226)	0.000
LAEF, %	0.843 (0.799–0.889)	0.000		
LASr, %	0.690 (0.623–0.764)	0.000	1.224 (1.085–1.381)	0.001
LASct, %	0.670 (0.597–0.751)	0.000		
E (cm/s)	1.028 (1.015–1.041)	0.000		
EDT (s)	0.984 (0.978–0.991)	0.000		
E‘ (sep) (cm/s)	0.857 (0.759–0.967)	0.013		
PASP (mmHg)	1.085 (1.049–1.123)	0.000		
E/e’	1.130 (1.058–1.206)	0.000	1.099 (1.004–1.203)	0.042
LAA parameters				
LAAEV, cm/s	0.913 (0.889–0.937)	0.000		
LAAFV, cm/s	0.942 (0.924–0.960)	0.000		
LAA ostia, mm	1.129 (1.056–1.207)	0.000		
LAA depth, mm	1.092 (1.048–1.137)	0.000	1.069 (1.010–1.130)	0.020
LAAEF, %	0.933 (0.917–0.949)	0.000	1.038 (1.013–1.063)	0.003

LVEF, Left ventricular ejection fraction; E, The early diastolic flow-velocity peak of mitral valve; (E'sep and E’la), early diastolic tissue Doppler velocity of mitral annulus from septal and lateral; E/e’, ratio of peak early mitral inflow velocity and peak early diastolic mitral annular velocity; PASP, pulmonary artery systolic pressure; LAAEV, the peak empty velocity of left atrial appendage; LAAFV, the peak filling velocity of left atrial appendage; LAA, left atrial appendage; LAAEF, Left atrial appendage ejection fraction; LARs, left atrial reservoir strain; LASct, left atrial strain conduit; LAVI, Left atrial volume index.

AUCs of CHADVASC2 score incorporated with LASr and LASr + CHADVASC2 were significantly higher than that of CHADVASC2 score (0.892 vs. 0.651, *P* < 0.001). Using a LASr cut-off value of <8.86% combined with CHADVASC2 cut-off value of 2 scores, patients with dense LAASEC were identified with a Sensitivity and specificity were 79% and 85%.

**Figure 4:**
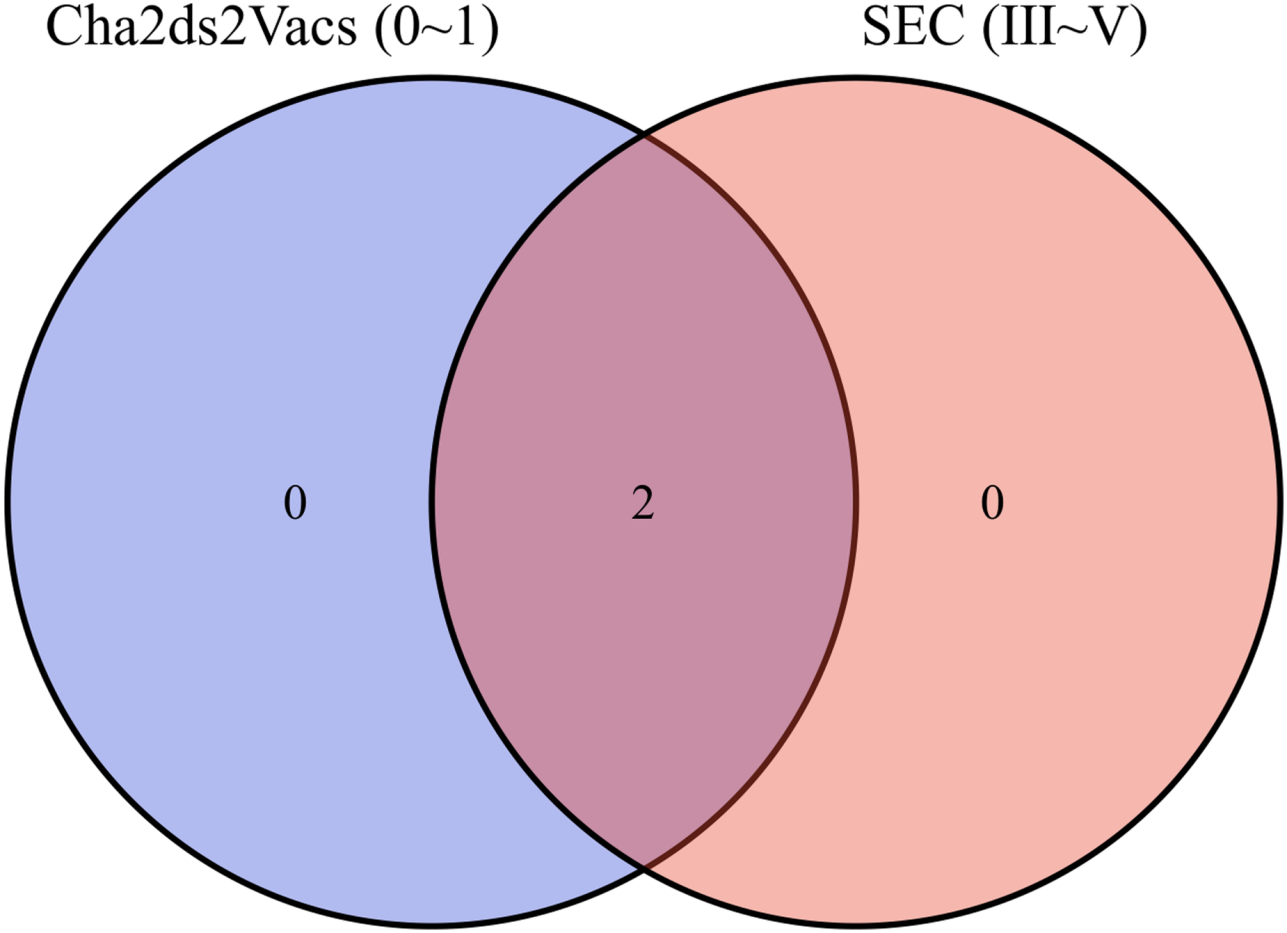
Venn diagram showed dense LAASEC intersection with the low anticoagulation score group.

Venn Diagram showed that there were dense SEC cases in the low anticoagulation score group (Figure 4).

**Figure 5:**
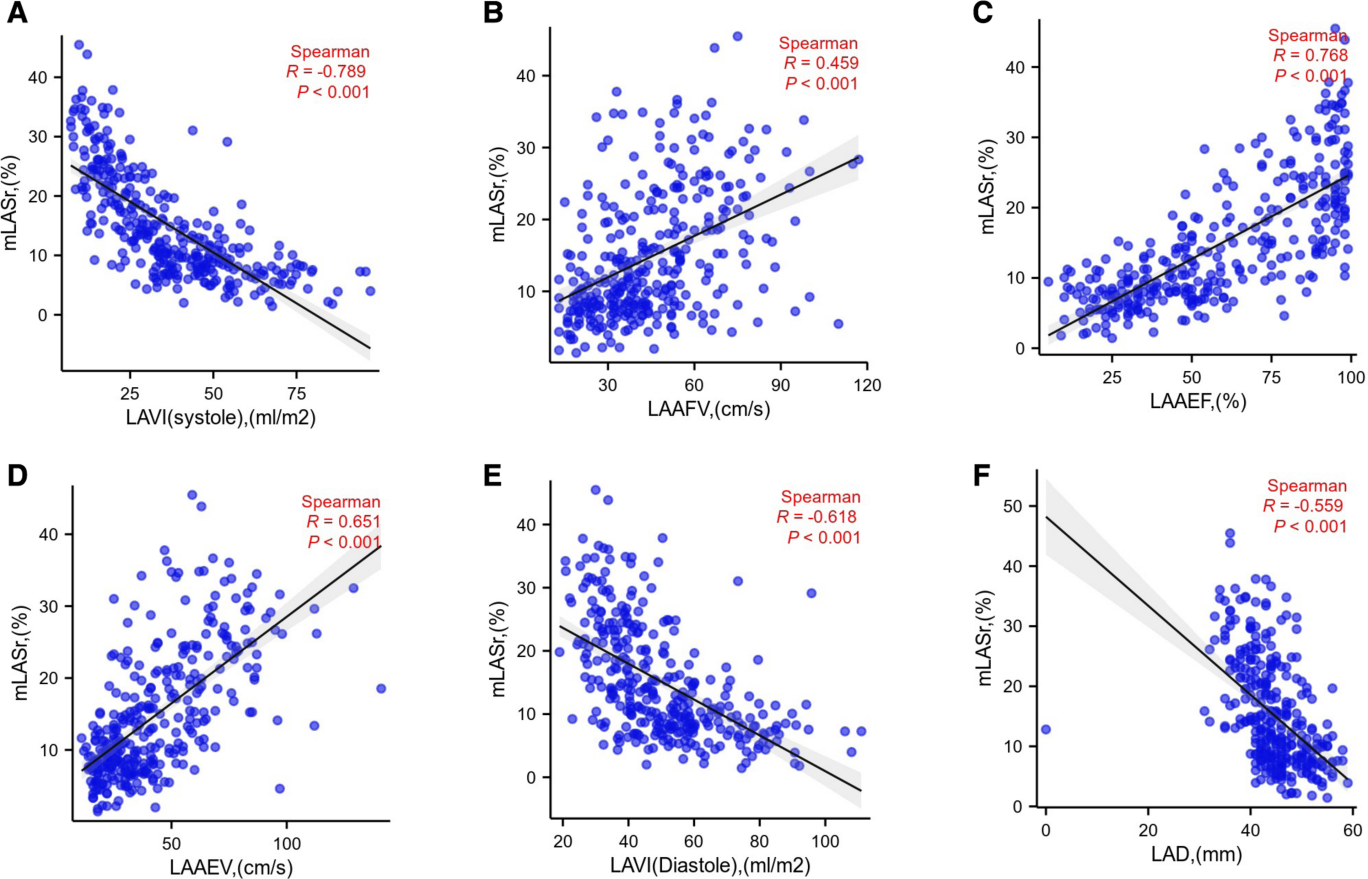
mLASr linear relationship with LAVI (systole), LAAFV, LAAEF, LAAEV, LAVI (diastole), LAD were displayed successively in (**A**), (**B**), (**C**), (**D**), (**E**), (**F**); LAD, Diameter of the left atrium; LASr, The left atrial peak longitudinal strain; LAVI , Left atrial volume index; LAAEV, the peak empty velocity of left atrial appendage; LAAFV, the peak filling velocity of left atrial appendage; LAA, left atrial appendage; LAAEF, Left atrial appendage ejection fraction.

(5) Linear relationship (Figure 5):

LASr is positively correlated with LAAEV, LAAFV, LAAEF, (*r* = 0.651, 0.459, 0.768, *P* < 0.001,repensively). LASr is negatively correlated with LAD, LAVI (diasoloe), LAVI (sysoloe) (*r* = −0.559, −0.618, −0.789 *P* < 0.001, repensively).

## 5. Discussion

The parameters in CHADVASC2 score are risk factors, which effect left atrial function. Normal aging involves the gradual loss of myocardial cells over time to replace fibrotic tissue. With the increase of age, the left atrium may become more rigid and its compliance may be reduced, for example, the left atrial reserve function may be reduced (13, 14). The synergistic effect of decreased left atrial reserve and ductal function may limit the effective volume transfer from left atrium to left ventricle (i.e., decreased left atrial emptying fraction (15). In particular, the left atrial ductal function of the elderly (≥70 years old) has almost halved compared with the individuals in their early 30 s, and the reserve function has seriously deteriorated compared with the same age range (16). Left atrial reserve function and ductal function are negatively correlated with age, which is consistent with previous reports (17).

The total strain and strain rate of left atrium in women were higher than those in men. In our current study, we have observed that, compared with men, the decline of left atrial function of female left atrial deformation indicators (especially left atrial reserve function) is more obvious with the increase of age. The overall strain damage of left atrium and the increase of left atrial volume index are both aging phenomena. Previous research results show that compared with aging men, women may have worse mechanical properties of left atrium (16). Deteriorated left atrial strain is considered to be closely related to motor ability (18). As the pathological feature of heart failure with preserved ejection fraction (19), this is a new public health problem worldwide. Although epidemiological studies have shown that heart failure with preserved ejection fraction is more common in elderly women, previous studies have shown that women are more likely to have left atrial mechanical dysfunction than men (16).

Previous reports provided evidence that elevated blood pressure may have a direct impact on left atrial mechanics (20–22). In addition, hypertension and diabetes have been proved to be independently related to the reduction of left atrial strain and left atrial strain rate, even if there is no change in left ventricle, which indicates that deformation measures are more sensitive to subtle changes in left atrial function before significant structural remodeling of hypertension (20). Consistent with their research results (16), our data showed that, regardless of the degree of left atrial remodeling, left atrial dysfunction was associated with high blood pressure value. However, regardless of the left ventricular diastolic index (NT proBNP or E/E′), the fact that this clinical left ventricular marker worsens with the increase of blood pressure may indicate that the increase of left ventricular end diastolic filling pressure cannot explain other mechanisms. We also observed that in our research participants, there was an inverse relationship between higher blood pressure and higher strain, which may be due to the need to enhance the left atrial pump pressurization function to resist the increase of left ventricular afterload, thus staying in the preclinical stage (23). Pellicori et al. showed that compared with patients without heart failure, patients with confirmed heart failure had more severe impairment of left atrial emptying function. Through multivariate Cox model, the increase of left atrial emptying capacity (rather than left ventricular ejection fraction) was independently related to the survival rate of patients with heart failure (24). Decreased (more severe) left atrial reserve was significantly associated with more advanced heart failure and more impaired left ventricular systolic and diastolic function indexes; The left atrial strain is superior to the left atrial reserve function measurement based on volume change; Impaired left atrial mechanical parameters strongly predict poor prognosis, independent of other clinical and echocardiographic prognostic factors (25). In sinus rhythm (SR) and atrial fibrillation (AF), heart failure (HF) with reduced left ventricular ejection fraction (LVEF) is associated with increased ischemic stroke and mortality. In patients with persistent nonvalvular atrial fibrillation, high left atrial asynchrony and impaired left atrial systolic and diastolic function may lead to a low flow state in the left atrial appendage (11, 26). Global peak atrial longitudinal strain was the best predictor of LAA thrombus and/or reduced LAA emptying in patients with persistent non-valvular AF (27). PALS has shown prognostic potential in LAAT identification in sinus rhythm patients (28). Impaired LA strain, as assessed by TTE, largely predicts LA thrombi by TEE in NVAF patients (29). Our conclusions are consistent with previous studies. The purpose of this study is to find the cardiogenic imaging risk indicators of pre-thrombotic state, echocardiogram parameters are still not used in day-to-day clinical practice as independent predictor for cardioembolic events in AF patients. Answering the current study question might provide an additional peace of data in this area.

Recent studies have shown that structural and functional abnormalities of the left atrium/left atrial appendage may also lead to stroke/transient ischemic attack. Enlargement of left atrial appendage, SEC and thrombus in left atrium/left atrial appendage, decreased emptying velocity of left atrial appendage (LAAEV) are recognized as stroke risk markers. Anatomical remodeling, atrial fibrosis and left atrial myocardial contractility decline are factors related to left atrial thrombosis/spontaneous echocardiography (LAT/SEC) and transesophageal echocardiography (TEE) reducing the flow rate of left atrial appendage (LAA). The decrease of LAA flow rate found by transesophageal echocardiography is likely to lead to stroke. The area of left atrial appendage (LAA) accounts for about 10% of the total left atrial volume and plays an important role in left atrial function.

With the development of ultrasound technology, researchers gradually understand the structure and function of left atrial appendage. The left atrial appendage (LAA) is a myogenic remnant of the left atrium, which can actively relax and contract. The left atrial appendage (LAA) has much stronger adaptability and blood storage capacity than other areas of the left atrium, so it is an important part of releasing the pressure of the left atrium and ensuring the filling of the left ventricle. Because of the complexity of structure and trabecular muscle, left atrial appendage is the most common site of left atrial thrombosis. Once atrial fibrillation occurs, the left atrium and left atrial appendage lose their contractility, resulting in the inability of blood to be effectively drained and detained, and eventually the SEC or thrombosis of the left atrial appendage is formed. The structural and functional remodeling of left atrium and left atrial appendage during AF, including cavity expansion, endocardial elastic fiber hyperplasia and myocardial function inhibition, are potential markers of left atrial appendage thrombosis (LAAT) and LAASEC (30–34). In this study, the LAA was larger and longer in the group of LAASEC grade 3 and above, and the left atrial appendage emptying fraction decreased, showing a relatively consistent situation with left atrial enlargement. The LAA is directly and closely connected with the left atrium. The LAA contributes a lot to regulating the left atrial pressure (volume and load). The increase of the left atrial pressure decreases the left atrial function, and on the contrary, it also affects the output of the LAA, causing the blood stasis of the left atrial appendage to show SEC.

The method was to measure the time interval between the start of *P* wave of ECG and the ejection of LAA (35). It can indirectly reflect the difference of cardiac atrial electrical conduction, PQ segment of ECG maybe correlations with mechanical and electrical activity dysfunction of left atrial. Since patients with per-AF are involved in the study and there is no *P* wave in their ECG, these connections cannot be considered temporarily. I hope to continue to consider them in the follow-up study.

## 6. Conclusion

Lower LASr assessed by 2D speckle-tracking echocardiography are significantly and associated with the dense LAASEC in patients with NVAF. The LASr may be superior to clinical and conventional echocardiography parameter for providing incremental value for risk stratification of the dense SEC.

## 7. Limitations

This study is a cross-sectional observational study, we hope to conduct longitudinal studies through long-term follow-up and observe if the ultrasound findings of this article in relation to clinical outcomes. Left atrial strain requires adequate apical view and good operation skills. It may be difficult to obtain the true atrial shape accurately, and it may be affected by the signal components generated by the structures around the left atrium. In patients with atrial fibrillation, the reliability and reproducibility of the left atrial longitudinal total strain is still lower than that in patients with normal sinus rhythm. As mentioned above, it needs to be measured in five cardiac cycles rather than three consecutive cardiac cycles.

Real time three-dimensional echocardiography is a non-invasive imaging method used to analyze the shape, volume and function of left atrium (LA), allowing simultaneous assessment of strain and volume changes frame by frame from 3D images. In addition, 3D speckle tracking echocardiography is becoming a clinical reality and is expected to become a clinical device for spatial analysis of local or global strain analysis of left atrium.

One of the most significant limitations is the patient population, as small samples may lack statistical capacity to demonstrate significant statistical differences.

## Data Availability

The original contributions presented in the study are included in the article, further inquiries can be directed to the corresponding authors.

## References

[1] Du Z Zhang B Lin M Tian Y Jing L Liu S The epidemiology of atrial fibrillation in Chinese postmenopausal women and its association with age of menopause. Maturitas. (2021) 143:151–6. 10.1016/j.maturitas.2020.10.010 33308621

[2] Wang Z Chen Z Wang X Zhang L Li S Tian Y The disease burden of atrial fibrillation in China from a national cross-sectional survey. Am J Cardiol. (2018) 122(5):793–8. 10.1016/j.amjcard.2018.05.015 30049467

[3] Saric M Armour AC Arnaout MS Chaudhry FA Grimm RA Kronzon I Guidelines for the use of echocardiography in the evaluation of a cardiac source of embolism. J Am Soc Echocardiogr. (2016) 29(1):1–42. 10.1016/j.echo.2015.09.011 26765302

[4] Peters DC. Association of left atrial fibrosis detected by delayed enhancement magnetic resonance imaging and risk of stroke in patients with atrial fibrillation. J Atr Fibrillation. (2011) 4(2):384. 10.4022/jafib.384 28496697 PMC5153052

[5] Di Biase L Santangeli P Anselmino M Mohanty P Salvetti I Gili S Does the left atrial appendage morphology correlate with the risk of stroke in patients with atrial fibrillation? Results from a multicenter study. J Am Coll Cardiol. (2012) 60(6):531–8. 10.1016/j.jacc.2012.04.032 22858289

[6] Kimura T Takatsuki S Inagawa K Katsumata Y Nishiyama T Nishiyama N Anatomical characteristics of the left atrial appendage in cardiogenic stroke with low CHADS2 scores. Heart Rhythm. (2013) 10(6):921–5. 10.1016/j.hrthm.2013.01.036 23384894

[7] Kim YG Choi JI Kim MN Cho DH Oh SK Kook H Non-vitamin K antagonist oral anticoagulants versus warfarin for the prevention of spontaneous echo-contrast and thrombus in patients with atrial fibrillation or flutter undergoing cardioversion: a trans-esophageal echocardiography study. PLoS One. (2018) 13(1):e0191648. 10.1371/journal.pone.0191648 29360845 PMC5779688

[8] Zhang E Liu T Li Z Zhao J Li G. High CHA2DS2-VASc score predicts left atrial thrombus or spontaneous echo contrast detected by transesophageal echocardiography. Int J Cardiol. (2015) 184:540–2. 10.1016/j.ijcard.2015.02.109 25767011

[9] Yao Y Shang MS Gao LJ Zhao JH Yang XH Liu T Elevated homocysteine increases the risk of left atrial/left atrial appendage thrombus in non-valvular atrial fibrillation with low CHA2DS2-VASc score. Europace. (2018) 20(7):1093–8. 10.1093/europace/eux189 28637244

[10] Zoghbi WA Adams D Bonow RO Enriquez-Sarano M Foster E Grayburn PA Recommendations for noninvasive evaluation of native valvular regurgitation: a report from the American society of echocardiography developed in collaboration with the society for cardiovascular magnetic resonance. J Am Soc Echocardiogr. (2017) 30(4):303–71. 10.1016/j.echo.2017.01.007 28314623

[11] Troughton RW Asher CR Klein AL. The role of echocardiography in atrial fibrillation and cardioversion. Heart. (2003) 89(12):1447–54. 10.1136/heart.89.12.1447 14617563 PMC1767994

[12] Lowe BS Kusunose K Motoki H Varr B Shrestha K Whitman C Prognostic significance of left atrial appendage “sludge” in patients with atrial fibrillation: a new transesophageal echocardiographic thromboembolic risk factor. J Am Soc Echocardiogr. (2014) 27(11):1176–83. 10.1016/j.echo.2014.08.016 25262162

[13] Hirsh BJ Copeland-Halperin RS Halperin JL. Fibrotic atrial cardiomyopathy, atrial fibrillation, and thromboembolism: mechanistic links and clinical inferences. J Am Coll Cardiol. (2015) 65(20):2239–51. 10.1016/j.jacc.2015.03.557 25998669

[14] Evin M Redheuil A Soulat G Perdrix L Ashrafpoor G Giron A Left atrial aging: a cardiac magnetic resonance feature-tracking study. Am J Physiol Heart Circ Physiol. (2016) 310(5):H542–9. 10.1152/ajpheart.00504.2015 26747498

[15] Boyd AC Schiller NB Leung D Ross DL Thomas L. Atrial dilation and altered function are mediated by age and diastolic function but not before the eighth decade. JACC Cardiovasc Imaging. (2011) 4(3):234–42. 10.1016/j.jcmg.2010.11.018 21414570

[16] Liao JN Chao TF Kuo JY Sung KT Tsai JP Lo CI Age, sex, and blood pressure-related influences on reference values of left atrial deformation and mechanics from a large-scale Asian population. Circ Cardiovasc Imaging. (2017) 10(10). 10.1161/CIRCIMAGING.116.006077 29021258

[17] Pathan F D'Elia N Nolan MT Marwick TH Negishi K. Normal ranges of left atrial strain by speckle-tracking echocardiography: a systematic review and meta-analysis. J Am Soc Echocardiogr. (2017) 30(1):59–70.e8. 10.1016/j.echo.2016.09.007 28341032

[18] Kraigher-Krainer E Shah AM Gupta DK Santos A Claggett B Pieske B Impaired systolic function by strain imaging in heart failure with preserved ejection fraction. J Am Coll Cardiol. (2014) 63(5):447–56. 10.1016/j.jacc.2013.09.052 24184245 PMC7195816

[19] Zsilinszka R Shrader P DeVore AD Hardy NC Mentz RJ Pang PS Sex differences in the management and outcomes of heart failure with preserved ejection fraction in patients presenting to the emergency department with acute heart failure. J Card Fail. (2016) 22(10):781–8. 10.1016/j.cardfail.2015.12.008 26687985

[20] Mondillo S Cameli M Caputo ML Lisi M Palmerini E Padeletti M Early detection of left atrial strain abnormalities by speckle-tracking in hypertensive and diabetic patients with normal left atrial size. J Am Soc Echocardiogr. (2011) 24(8):898–908. 10.1016/j.echo.2011.04.014 21665431

[21] Kokubu N Yuda S Tsuchihashi K Hashimoto A Nakata T Miura T Noninvasive assessment of left atrial function by strain rate imaging in patients with hypertension: a possible beneficial effect of renin-angiotensin system inhibition on left atrial function. Hypertens Res. (2007) 30(1):13–21. 10.1291/hypres.30.13 17460367

[22] Eshoo S Boyd AC Ross DL Marwick TH Thomas L. Strain rate evaluation of phasic atrial function in hypertension. Heart. (2009) 95(14):1184–91. 10.1136/hrt.2008.156208 19398436

[23] Klein AL Burstow DJ Tajik AJ Zachariah PK Bailey KR Seward JB. Effects of age on left ventricular dimensions and filling dynamics in 117 normal persons. Mayo Clin Proc. (1994) 69(3):212–24. 10.1016/S0025-6196(12)61059-3 8133658

[24] Pellicori P Zhang J Lukaschuk E Joseph AC Bourantas CV Loh H Left atrial function measured by cardiac magnetic resonance imaging in patients with heart failure: clinical associations and prognostic value. Eur Heart J. (2015) 36(12):733–42. 10.1093/eurheartj/ehu405 25336215

[25] Carluccio E Biagioli P Mengoni A Francesca Cerasa M Lauciello R Zuchi C Left atrial reservoir function and outcome in heart failure with reduced ejection fraction. Circ Cardiovasc Imaging. (2018) 11(11):e007696. 10.1161/CIRCIMAGING.118.007696 30571318

[26] Zabalgoitia M Halperin JL Pearce LA Blackshear JL Asinger RW Hart RG. Transesophageal echocardiographic correlates of clinical risk of thromboembolism in nonvalvular atrial fibrillation. Stroke prevention in atrial fibrillation III investigators. J Am Coll Cardiol. (1998) 31(7):1622–6. 10.1016/S0735-1097(98)00146-6 9626843

[27] Cameli M Lunghetti S Mandoli GE Righini FM Lisi M Curci V Left atrial strain predicts pro-thrombotic state in patients with non-valvular atrial fibrillation. J Atr Fibrillation. (2017) 10(4):1641. 10.4022/jafib.1641 29487679 PMC5821630

[28] Kurzawski J Janion-Sadowska A Zandecki L Piatek L Koziel D Sadowski M. Global peak left atrial longitudinal strain assessed by transthoracic echocardiography is a good predictor of left atrial appendage thrombus in patients in sinus rhythm with heart failure and very low ejection fraction - an observational study. Cardiovasc Ultrasound. (2020) 18(1):7. 10.1186/s12947-020-00188-0 32061249 PMC7024551

[29] Sonaglioni A Lombardo M Nicolosi GL Gensini GF Ambrosio G. Mechanical concordance between left atrium and left atrial appendage in nonvalvular atrial fibrillation: can it be exploited to avoid transesophageal echocardiography prior to electrical cardioversion during COVID-19 pandemic? Int J Cardiovasc Imaging. (2022) 38(2):351–62. 10.1007/s10554-021-02414-w 34537932 PMC8449702

[30] Fatkin D Kelly RP Feneley MP. Relations between left atrial appendage blood flow velocity, spontaneous echocardiographic contrast and thromboembolic risk in vivo. J Am Coll Cardiol. (1994) 23(4):961–9. 10.1016/0735-1097(94)90644-0 8106703

[31] Ayirala S Kumar S O’Sullivan DM Silverman DI. Echocardiographic predictors of left atrial appendage thrombus formation. J Am Soc Echocardiogr. (2011) 24(5):499–505. 10.1016/j.echo.2011.02.010 21440414

[32] Akoum N Fernandez G Wilson B McGann C Kholmovski E Marrouche N. Association of atrial fibrosis quantified using LGE-MRI with atrial appendage thrombus and spontaneous contrast on transesophageal echocardiography in patients with atrial fibrillation. J Cardiovasc Electrophysiol. (2013) 24(10):1104–9. 10.1111/jce.12199 23844972 PMC3818287

[33] Shirani J Alaeddini J. Structural remodeling of the left atrial appendage in patients with chronic non-valvular atrial fibrillation: implications for thrombus formation, systemic embolism, and assessment by transesophageal echocardiography. Cardiovasc Pathol. (2000) 9(2):95–101. 10.1016/S1054-8807(00)00030-2 10867359

[34] Ma N Lu R Zhao D Jiang Z Tang M Bao C Left atrial appendage fibrosis and 3-year clinical outcomes in atrial fibrillation after endoscopic ablation: a histologic analysis. Ann Thorac Surg. (2020) 109(1):69–76. 10.1016/j.athoracsur.2019.05.055 31302082

[35] Kinay O Nazli C Ergene O Dogan A Gedikli O Hoscan Y Time interval from the initiation of the electrocardiographic P wave to the start of left atrial appendage ejection flow: a novel method for predicting atrial fibrillation recurrence. J Am Soc Echocardiogr. (2002) 15(12):1479–84. 10.1067/mje.2002.127515 12464915

